# Sagittal spino-pelvic parameters of adult isthmic spondylolisthesis

**DOI:** 10.1186/1748-7161-10-S1-P18

**Published:** 2015-01-19

**Authors:** Feng Zhu, Hongda Bao, Shouyu He, Zezhang Zhu, Yong Qiu, Xu Sun, Zhen Liu, Bin Wang

**Affiliations:** 1 Spine Surgery, Affiliated Drum Tower Hospital of Nanjing University Medical School, China

## Objective

To compare the differences of spino-pelvic parameters between patients with isthmic spondylolisthesis of different grades, and to investigate the correlation between L5 incidence angle (L5I) and the percentage of spondylolisthesis.

## Methods

60 patients with L5-S1 isthmic spondylolisthesis (mean age, 47years, range, 28 to 69 years) and age-matched control group of 77 normal adults (mean age, 43.5 years, range, 25 to 63 years) were recruted in this retrospective study. Parameters including slip distance (SD), slipping percentage (SP), pelvic incidence (PI), pelvic tilt (PT), sacral slope (SS), L5 incidence (L5I), lumbar-sacral angle (LSA), lumbar lordosis (LL), sagittal vertical axis (SVA) were measured on the long-cassette standing upright lateral radiographs of the spine and pelvis. Patients with spondylolisthesis were divided into two groups based on slipping percentage : Low grade : group A with SP<30percent (30 cases of 60) and high grade: group B with SP >30percent (30 cases of 60). Differences in sagittal parameters among groups were analyzed using independent samples t-test, and Pearsons correlation coefficients were used to investigate the relationship between spino-pelvic parameters and SP.

## Result

PI, PT, SS, and LL are larger (p<0.05) in subjects with isthmic spondylolisthesis than those in the control group, while LSA is significantly decreased. L5I is significantly greater in group B, as compared with control group, while there was no significant difference between group A and control group. Strong positive correlation between the SP and PI, PT, SS, SVA and negative correlation between SP and LSA were confirmed in all the patients with spondylolisthesis. SP shows a positive correlation with L5I in group B(p<0.05). However, SP presents no statistically significant correlation with L5I in group A. L5I demonstrates a positive correlation with PI and shows a inverse relation to LSA in the total isthmic spondylolisthesis group.

## Conclusion

Spino-pelvic parameters including PI, PT, SS and LL were significantly greater in adult patients with isthmic spondylolisthesis. L5I significantly increased in patients with relatively severer spondylolisthesis and showed postive correlation with slipping percentage in those patients which indicated that L5I can be treated as a risk factor that correlated with the development of spondylolisthesis. Then, more attention should be paid to the improvement of L5I for patients with relatively severer spondylolisthesis.

